# An Association Between Phonetic Complexity of Infant Vocalizations and Parent Vowel Hyperarticulation

**DOI:** 10.3389/fpsyg.2021.693866

**Published:** 2021-07-19

**Authors:** Ellen Marklund, Ulrika Marklund, Lisa Gustavsson

**Affiliations:** ^1^Phonetics Laboratory, Stockholm Babylab, Department of Linguistics, Stockholm University, Stockholm, Sweden; ^2^Division of Sensory Organs and Communication, Department of Biomedical and Clinical Sciences, Linköping University, Linköping, Sweden; ^3^Speech and Language Clinic, Department of Neurology, Danderyd Hospital, Stockholm, Sweden; ^4^Division of Speech and Language Pathology, Department of Clinical Science, Intervention and Technology, Karolinska Institutet, Stockholm, Sweden

**Keywords:** vowel hyperarticulation, vowel space area, infant-directed speech, word-complexity measure for Swedish, WCM-SE, phonetic complexity, communicative adaptiveness, parent-infant interaction

## Abstract

Extreme or exaggerated articulation of vowels, or vowel hyperarticulation, is a characteristic commonly found in infant-directed speech (IDS). High degrees of vowel hyperarticulation in parent IDS has been tied to better speech sound category development and bigger vocabulary size in infants. In the present study, the relationship between vowel hyperarticulation in Swedish IDS to 12-month-old and phonetic complexity of infant vocalizations is investigated. Articulatory adaptation toward hyperarticulation is quantified as difference in vowel space area between IDS and adult-directed speech (ADS). Phonetic complexity is estimated using the Word Complexity Measure for Swedish (WCM-SE). The results show that vowels in IDS was more hyperarticulated than vowels in ADS, and that parents’ articulatory adaptation in terms of hyperarticulation correlates with phonetic complexity of infant vocalizations. This can be explained either by the parents’ articulatory behavior impacting the infants’ vocalization behavior, the infants’ social and communicative cues eliciting hyperarticulation in the parents’ speech, or the two variables being impacted by a third, underlying variable such as parents’ general communicative adaptiveness.

## Introduction

Infant-directed speech (IDS), the speech style commonly used when speaking to infants and small children (e.g., Soderstrom, 2007; Golinkoff et al., 2015), has been reported to facilitate or promote various aspects of early language development (e.g., Trainor and Desjardins, 2002; Singh et al., 2009; Ma et al., 2011; Bosseler et al., 2016; Foursha-Stevenson et al., 2017). One specific characteristic commonly found in IDS, clear or exaggerated vowel articulation (for a review, see Marklund and Gustavsson, 2020) compared to adult-directed speech (ADS), has been linked to better speech sound discrimination (Liu et al., 2003; García-Sierra et al., 2021), better word-recognition (Song et al., 2010), as well as larger receptive and productive vocabulary sizes (Hartman et al., 2017; Kalashnikova and Burnham, 2018). The present study focuses on the relationship between parents’ articulation and children’s own productions. Since phonetic detail of parent vocalizations can directly impact the phonetic form of child vocalizations (Goldstein and Schwade, 2008), it is hypothesized that a relationship will be found between degree of parents’ vowel hyperarticulation and phonetic complexity of infant vocalizations.

### Vowel Hyperarticulation and Language Development

The way in which speech is realized is highly variable. Speakers adapt their articulation to the perceived demands of the listener (Lindblom, 1990), for example speaking more clearly in noisy environments (Šimko et al., 2016) or using more reductions when uttering semantically predictable words than when uttering unpredictable words (Clopper and Pierrehumbert, 2008). These adaptations mean that individual speech sounds are realized on a continuum that ranges from exaggerated articulation (i.e., hyperarticulation) to relaxed articulation (i.e., hypoarticulation). IDS has been reported to be more hyperarticulated overall than ADS in some cases (vowels: Kuhl et al., 1997; see Marklund and Gustavsson, 2020 for a review; consonants: Cristià, 2010; McMurray et al., 2013) and more hypoarticulated than ADS overall in other cases (vowels: Englund and Behne, 2005, 2006; Benders, 2013; Englund, 2018; consonants: Sundberg and Lacerda, 1999; Benders et al., 2019). In this study, the focus is articulatory adaptation of vowels, and it will be referred to as vowel hyperarticulation (VH), since vowels in IDS to Swedish 12-month-old infants has previously been shown to be more hyperarticulated overall than vowels in Swedish ADS (Marklund and Gustavsson, 2020).

Vowel hyperarticulation has been suggested to promote infant learning of speech sound categories by exaggerating the distance between different vowel categories (e.g., Bernstein Ratner, 1984; Kuhl et al., 1997). However, detailed investigation of variability within and between different vowel categories in IDS contra in ADS suggests instead that IDS provides higher within-category variability than ADS (Cristia and Seidl, 2014), which, based on findings from second language learning, might contribute to more robust speech sound categories (Wong, 2014). Testing the relationship between VH in parent IDS and infant speech sound perception, a positive correlational relationship has been reported. Specifically, more hyperarticulation in mothers’ IDS correlates with better discrimination of a native fricative contrast in Mandarin-learning 6–8-month-old and 10–12-month-old infants (Liu et al., 2003). Similar findings have been reported for English-Spanish bilingual infants at 11 and 14 months, but only for a native Spanish contrast, not a non-native Mandarin contrast (García-Sierra et al., 2021).

Vowel hyperarticulation in mothers’ IDS also predicts infant vocabulary. In a longitudinal study, Australian English IDS to infants from 9 months and up predicted parent-reported expressive vocabulary size at ages 15 and 19 months (Kalashnikova and Burnham, 2018). Similarly, American English-learning children, whose mothers speak to them with high degrees of VH at 18 months, score higher in standardized vocabulary tests at 24 months than do children of mothers who use less hyperarticulation (Hartman et al., 2017). One potential explanation for an impact of VH on vocabulary size is that it helps infants more readily identify the words they hear. In favor of that notion, 19-month-old infants recognize known words faster when they are spoken with hyperarticulated vowels than when they are not (Song et al., 2010).

To summarize, previous research has reported a positive relationship between VH and other language skills/outcomes, specifically speech sound category perception (Liu et al., 2003; García-Sierra et al., 2021) and vocabulary development (Hartman et al., 2017; Kalashnikova and Burnham, 2018). This study expands the scope of language skills investigated in relation to VH to that of infants’ own productions.

### Parent Feedback and Infant Vocalizations

Infant vocalizations are influenced by parent social and vocal behavior. For example, removing social feedback from adult-infant interaction leads to fewer vocalizations from 5-month-old infants (Goldstein et al., 2009), and providing social feedback in response to 8-month-old’s vocalizations results both in a higher number of vocalizations and more mature vocalizations (syllabic rather than vocalic, more canonical syllables, fully voiced) compared to social feedback non-contingent to infant productions (Goldstein et al., 2003). When it comes to vocal behavior, amount of parent IDS in parent-infant interactions correlates with amount of infant speech output (Dunst et al., 2012; Ramírez-Esparza et al., 2014; Spinelli et al., 2017). Vocal feedback from adults also influences the phonetic form of infant vocalizations. Just the presence of contingent vocal feedback leads to a higher ratio of mature vocalizations (syllabic rather than vocalic) in 3-month-olds (Bloom et al., 1987). In interactions between mothers and their 9.5-month-olds, the specific type of vocal feedback infants received impacted their vocal production. Infants whose mothers responded to vocalizations with a long vowel sound produced more fully voiced vocalizations, while infants whose mothers responded with words produced more consonant-vowel syllables (Goldstein and Schwade, 2008).

These findings demonstrate that the phonetic content of parent utterances can have an impact on the phonetic realization of infant vocalizations. This study therefore investigates whether degree of parent VH in IDS correlates with phonetic complexity of infant vocalizations.

### This Study

In the present study, parents’ articulatory adaptations in terms of VH is quantified using the difference in of vowel space area (VSA), that is, the area between the point vowel formant means in *F*_1_-*F*_2_ space, between IDS and ADS (Kuhl et al., 1997; Marklund and Gustavsson, 2020). Phonetic complexity of children’s productions is measured using the Swedish adaptation of the Word-Complexity Measure (WCM-SE). This measure assigns a complexity score to each vocalization based on number of syllables, stress position, as well as number, position and combinations of articulatory complex speech sounds (Marklund et al., 2018). To determine whether VH could be established in the present IDS sample, the VSA was compared between IDS and ADS. To investigate whether there is a link between parents’ articulatory adaptations and phonetic complexity of infants’ vocalization, the relationship between VSA difference between IDS and ADS, and infants’ mean WCM-SE score, was tested for correlation.

## Materials and Methods

### Participants

Nineteen infants (mean age = 12 months, range = 11.5--12.3, SD = 0.2) and their parents participated in the study (9 girls, 10 boys; 12 mothers, 7 fathers). All infants were born full-term (within 3 weeks of due date) and monolingual (defined as both parents speaking only Swedish with the infant). The majority of the parents (*n* = 15) had university education, and all had completed high school (which entails three non-obligatory years of education after the mandatory 9--10 years of basic education in Sweden). The participants constitute a subset of a larger group of subjects (*n* = 72), taking part in a longitudinal study in which parent-child interactions were recorded every 3--6 months, when the child was between 3 months and 3 years^1^. Free-play interaction sessions were recorded at each lab-visit, but additional tests and tasks varied between visits. An ADS sample was recorded at the 27-month-old visit. Inclusion criteria for participants in the present study were (a) there was a recording from the 12-month visit, (b) the infant was monolingual, and (c) there was sufficient ADS material from the same parent as in the 12-month visit.

The study was approved by the Regional Ethics Review Board (2015/63-31). Recruitment of infants in the appropriate age and their parents living in the greater Stockholm area was conducted via mail with invitation to participate in the study. Addresses were obtained via the Swedish Tax Agency. Parents received memory-sticks with all their audio and video recordings as thanks for their participation in the longitudinal study.

### Recordings

Audio and video recordings of parent-infant interaction were made at Stockholm Babylab, the Phonetics Laboratory, Stockholm University in a comfortable carpeted studio equipped with age-appropriate furniture and toys. The recordings were made with three wall mounted cameras (Canon XA10) to capture all angles of the parent interacting with the infant. A fourth camera (GoPro Hero3), attached to the parent’s chest, enabled close up video uptake of the infant from the parent perspective. To capture high-quality audio, omni-directional wireless lavalier microphones (Sennheiser EW 100 G2) were mounted on parent and infant, and one room microphone (AKG SE 300 B) was mounted on a high shelf. In the present study, audio from the lavalier microphones was used, since this setup enabled high-quality close-up recordings of the adult’s speech and the infant’s vocalizations with minimal interference from the other speaker.

The recording sessions lasted for approximately 10 min. The experimenter instructed parents to interact, play and talk with their infant as they typically would at home. After instructions and equipment arrangements, the researchers left the studio and monitored the recording from the adjacent control room. For the ADS material, a conversation between the parent and the experimenter about the infant and their participation in the study was recorded. This recording took place at the beginning of the lab visit when the infant was around 27 months old. Parents were encouraged to speak as much and as freely as possible, and the conversation typically lasted around 2–5 min.

### Vowel Hyperarticulation Estimations in Parent Speech

Estimation of VH in parent’s IDS and ADS was performed as a part of a previous study, and detailed information about the procedure can be found there (Marklund and Gustavsson, 2020). In brief, parent speech was quasi-orthographically transcribed using ELAN 4.6.2-5.3 (Sloetjes and Wittenburg, 2008). The transcriptions (for annotation protocol see Gerholm, 2018) were automatically segmented, converted to IPA and aligned with their audio files using the web service WebMAUS General 5.33 of the Bavarian Archive for Speech Signals at the University of Munich (Schiel, 1999; Kisler et al., 2017).

Formants were estimated for the audio recordings using Praat 6.0.37 and 6.0.40 (Boersma and Weenink, 2018). Default settings were applied, except for formant ceiling and maximum number of expected formants (Escudero et al., 2009). Since reliability of formant estimations decreases with higher fundamental frequency (*f*_o_), vowels with a median *f*_o_ exceeding 350 Hz were excluded (Lindblom, 1962; Monsen and Engebretson, 1983). To reduce the impact of coarticulation, the mid 40% of the vowel was used in the analysis (Marklund and Gustavsson, 2020). Each parents’ average *F*_1_ and *F*_2_ for the point vowels /i/, /ɑ/, and /u/ in IDS and ADS were extracted and used for calculations of VSA. VSA was calculated in R 3.5.0 (R Core Team, 2018), separately for IDS (VSA_IDS_) and ADS (VSA_ADS_), using the following formula (Liu et al., 2003):

(1) $$ABS\left(\left(iF_{1}\;\times \;\left(aF_{2}\;-\;uF_{2}\right)\;+\;aF_{1}\;\times \;\left(uF_{2}\;-\;iF_{2}\right)\;+\;uF_{1}\;\times \;\left(iF_{2}\;-\;aF_{2}\right)\right)\;/\;2\right)$$

This study uses the difference in VSA between IDS and ADS as the measure of parents’ tendency to adapt their articulation when speaking to infants.

### Phonetic Complexity Estimations of Infant Vocalizations

**FIGURE 1:**
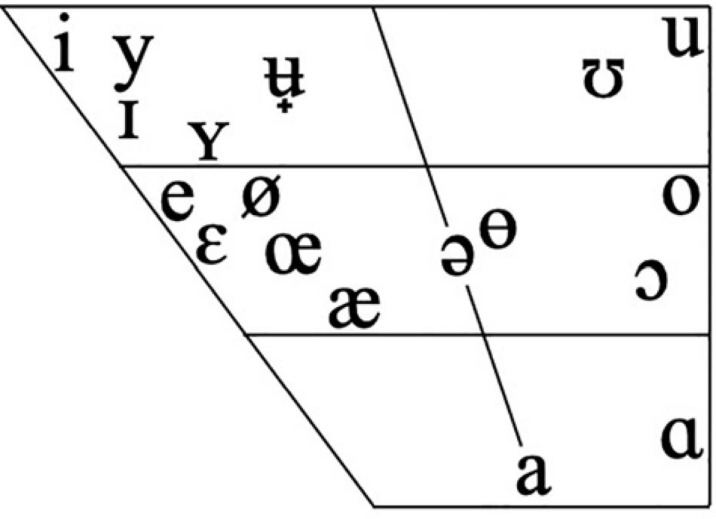
The Swedish vowels used in the transcription of infant vocalizations. Figure adapted from IPA Chart from International Phonetic Association to include phonemes specified in Engstrand (1999).

Infant vocalizations were phonetically transcribed in ELAN 5.8–5.9 (Sloetjes and Wittenburg, 2008) by two experienced phoneticians (authors UM and LG) according to an annotation protocol developed for compatibility with WCM-SE (Marklund et al., 2018). The protocol entailed transcribing all sounds present in the Swedish phoneme inventory as described in Engstrand (1999), with the addition of a number of other common allophones (Table 1 and Figure 1). Segments not recognizable as any of those phonemes were marked as “C” (if consonant-like) or “V” (if vowel-like) in the transcription. If not possible to determine whether the sound was a consonant or a vowel, it was denoted by a square. Syllable boundaries and primary stress were also marked up in each vocalization. Boundaries between vocalizations were based on silence (pause or breath), and thus not dependent on semantic interpretation. All infant vocalizations consisting of words, syllables, babbling or isolated speech sounds were transcribed. Overlapping or distorted speech, laughter, crying, fuzzing, coughing, effort sounds and vegetative sounds such as breathing, sneezes and hiccups were excluded.

**TABLE 1:** The Swedish consonants used in the transcription of infant vocalizations.

	**Bilabial**		**Labiodental**		**Dental**		**Retroflex**		**Alveolar**		**Palatal^†^**		**Velar**		**Uvular**		**Glottal**	
Plosive	p	b			t	d	t	ɖ					k	g				
Nasal		m				n		ɳ						ŋ				
Trill						r										r		
Tap/flap						ɾ												
Fricative			f	v	s		ʂ	ʐ				ʝ				ʁ		h
Approximant										ɹ								
Lat. Approximant						l		ɭ										

*^†^Also /ɧ/ (voiceless dorso-palatal/velar fricative) and /ɕ/ (voiceless alveolo-palatal fricative). Table adapted from IPA Chart from International Phonetic Association to include phonemes specified in Engstrand (1999).*

Two recordings were transcribed by both annotators independently to check inter-transcriber agreement. Percentage of matching characters for each transcribed vocalization was compared. Inter-transcriber agreement of which vocalizations were transcribed was 70% and out of those the average transcription inter-transcriber agreement was 78%.

To estimate complexity in infant vocalizations, the WCM-SE was used (Marklund et al., 2018). Based on a number of phonological/phonetic complexity parameters in three domains, a complexity score is calculated for each vocalization based on how many of the parameters are present in it (Table 2). For example, the Swedish word *elefant* (“elephant”) produced as /ele'fant/ results in a WCM-SE score of 6, the word *sko* (“shoe”) produced as /sku:/ results in a score of 3, and the word *mamma* (“mommy”) produced as /màma/ results in a score of 0. WCM-SE points were calculated for each vocalization based on the transcriptions using a script written in R 3.5.0–4.0.2 (R Core Team, 2018). Although the WCM-SE is originally a phonological measure, the principles parameters are based on phonetic complexity (Marklund et al., 2018), so it can also be used for estimation of phonetic complexity.

**TABLE 2:** The WCM-SE measure as implemented in the present study, based on Marklund et al. (2018).

**Domains**	**Complexity parameter**	**N points**
Word patterns	>2 syllables	1 per vocalization
	Non-initial stress	1 per vocalization
Syllable structures	Word-final consonant	1 per vocalization
	Consonant cluster^†^	1 per occurrence
Sound classes	Velar consonant [k], [g], [**ŋ**], [**ɧ**]	1 per occurrence
	Liquid [l], [**ɭ**], [**ɹ**]	1 per occurrence
	Fricative^‡^ [f], [v], [s], [**ʐ**], [**ʁ**], [**ʂ**], [**ʝ**], [h], [**ɧ**], [**ɕ**]	1 per occurrence
	Voiced fricative [v], [**ʐ**], [**ʁ**], [**ʝ**]	1 per occurrence
	Trill [r], [r]	3 per occurrence
	Long, front, rounded vowel [y], [ø], 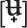	1 per occurrence

*Each transcribed vocalization was given a WCM-SE score, after which a subjectmean score was calculated.*

*^†^Only consonant clusters within syllables were counted.*

*^‡^Vocalization-final [h] excluded, since that is likely to be release of breath.*

### Analyses

In order to establish that IDS vowels were hyperarticulated relative to ADS vowels, parents’ VSA_IDS_ and VSA_ADS_ were compared. Second, it was tested whether parents’ adaptation in terms of hyperarticulation, described as VSA difference (VSA_IDS_–VSA_ADS_), predicted infants’ average WCM-SE scores. The analysis was performed in R 3.5.0 (R Core Team, 2018).

## Results

**FIGURE 2:**
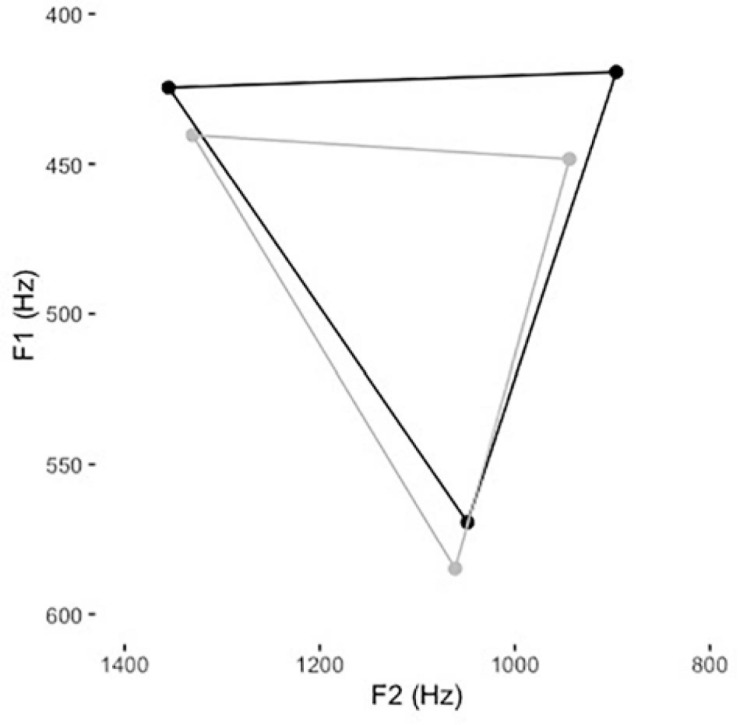
VSAs for IDS (black) and ADS (gray), based on */i/* (top left dots), */a/* (bottom dots), and */u/* (top right dots).

Formant estimations for point vowels in IDS and ADS can be found in Table 3 and Figure 2.

**TABLE 3:** Descriptive statistics of formant estimations of point vowels in IDS and ADS.

**Speech sample**	**Vowel type**	**Mean *F*_1_ (SD) in Hz**	**Mean *F*_2_ (SD) in Hz**	**N included tokens**
IDS	i	453 (88)	1,972 (305)	507
	u	430 (90)	934 (195)	223
	ɑ	656 (117)	1,383 (263)	936
ADS	i	472 (81)	1,904 (272)	282
	u	473 (75)	1,032 (229)	119
	ɑ	661 (96)	1,307 (234)	667

A two-tailed paired-samples *t*-test revealed a significantly larger VSA in IDS than in ADS [*t*(18) = 3.87, *p* = 0.001], with a mean of 101,021 Hz^2^ in IDS (SD = 35,831) and 74,304 Hz^2^ in ADS (SD = 35,639).

**FIGURE 3:**
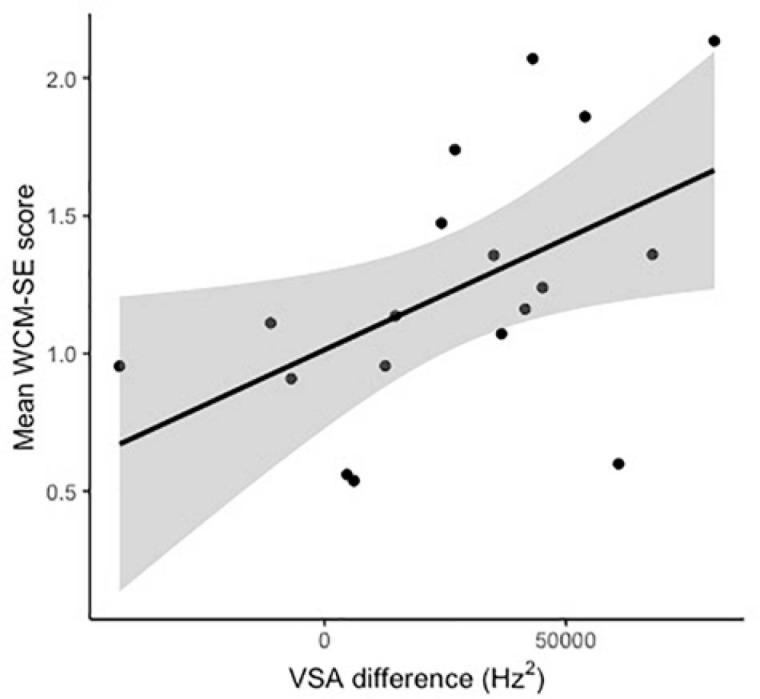
Scatter plot showing the relationship between VSA difference (VSA_IDS_–VSA_ADS_) and WCM-SE score for individual participants.

Subjects’ mean WCM-SE scores ranged from 0.54 to 2.13 (mean = 1.23, SD = 0.48, after excluding one outlier with a score of 4.69). The mean VSA difference of the included participants was 27,405 Hz^2^ (SD = 30,810). A linear regression showed that VSA difference significantly predicted WCM-SE score [*F*(1,16) = 5.89, β = 8.07e-6, *R*^2^ = 0.223, *p* = 0.027], see Figure 3. For a 10000 Hz^2^ increase in VSA difference, the model predicts an 0.08 increase in WCM-SE score.

Frequencies are reported in Hz for the results to be comparable with previous similar studies (Liu et al., 2003; García-Sierra et al., 2021), however, the same pattern of results was found when converting the Hz values to Bark (Winkelmann et al., 2021) for each vowel token prior to calculating the VSA [*t*-test: *t*(18) = 3.75, *p* = 0.001; linear regression: *F*(1,16) = 5.89, β = 0.19, *R*^2^ = 0.228, *p* = 0.045].

## Discussion

Swedish IDS to 12-month-old infants show more VH than ADS, in terms of VSA. Difference in hyperarticulation between IDS and ADS, that is, parents’ degree of articulatory adaptation in terms of hyperarticulation, correlates positively with phonetic complexity of infant vocalizations.

Previous studies have reported VH in IDS to infants around 1 year of age, both in Swedish (Marklund and Gustavsson, 2020)^2^ and in IDS of several other languages (e.g., Liu et al., 2003; Kondaurova et al., 2012; Kalashnikova et al., 2017, 2018; Tang et al., 2017; Kalashnikova and Burnham, 2018). However, IDS to Dutch-learning 11-month-old instead report hypoarticulation in IDS compared to in ADS (Benders, 2013), and in some cases, no difference was found between IDS and ADS (Dodane and Al-Tamimi, 2007; Kondaurova et al., 2012; Xu et al., 2013; Burnham et al., 2015). These disparate findings suggest that the occurrence of VH in IDS might be dependent on context more fine-grained than language and infant age. For example, in conversations between adults, more hyperarticulation is found in rare words than in more common words (Munson and Solomon, 2004). This means that parents potentially adapt their articulation to different degrees for different words, based on expectations of whether or not their child understands them.

Positive relationships between VH and different language skills and outcomes have previously been reported, specifically speech sound category development (Liu et al., 2003; García-Sierra et al., 2021) and vocabulary (Hartman et al., 2017; Kalashnikova and Burnham, 2018). The present findings add phonetic complexity of vocalizations to the list. Just like the previous studies, the present study does not provide information about causality. Although one study has shown that it is possible for parent articulatory behavior to impact infant vocalizations (Goldstein and Schwade, 2008), it is equally possible for parent VH to be elicited by interactional cues from the infant. When mothers interact with their infant without the infant being able to hear them, they do not hyperarticulate their vowels, indicating that their articulatory adaptation is a response to in-the-moment cues from the infant (Lam and Kitamura, 2012). It might also be the case that there is no causal relationship between parent VH and the various language skills and outcomes of the child studied so far, but that they are instead impacted by other, underlying, variables. One possible such underlying variable is general communicative adaptiveness of the parent. Articulatory adaptation can be considered a specific type of realization of general communicative adaptiveness. Other types of communicative adaptive behaviors include temporal and conceptual contingency as well as prosodic entrainment. One broad concept that captures communicative adaptation, parent responsiveness (either in terms of a general sensitivity to the infant’s communicative needs or in terms of contingent responses), has previously been linked to speech sound category development (Elsabbagh et al., 2013), vocabulary development (Tamis-LeMonda et al., 2014; Marklund et al., 2015), and maturity of infant vocalizations (Bloom et al., 1987; Goldstein et al., 2003). All of these behaviors, VH, parent responsiveness, a propensity to provide temporally and conceptually contingent responses, as well as prosodic entrainment, may well be different realizations of the same underlying communicative adaptiveness, which could be beneficial to infant language development and/or an inherited trait.

Limitations in this study include a relatively small sample size, although in line with previous similar studies (Liu et al., 2003; Hartman et al., 2017; Kalashnikova and Burnham, 2018; García-Sierra et al., 2021). Further, the WCM-SE measure is originally intended for assessment of phonological rather than phonetic complexity, and the infants in this study are too young to be expected to have a phonological system fully in place. However, the WCM-SE is based on phonetic principles, and can therefore be used independently of phonological development. The rationale for using WCM-SE specifically is that previous findings have shown that the phonetic content of parents’ utterances impacts the phonetic content of infant vocalizations (Goldstein and Schwade, 2008), and WCM-SE is more informative about the infants’ articulation than other, less detailed estimates of infant production (number of CV syllables, etc.).

The present study contributes to the field of language development by discussing VH in IDS in the context of broader phonetic and speech communication principles. Rather than viewing VH as a characteristic specific to IDS, it is here treated as part of speech communication in general, and this perspective is applied to the discussion on how it relates to early language development. From this perspective, future studies on VH in IDS should focus on variations in the more fine-grained linguistic context, in order to provide clarity on why it is realized the way it is in IDS compared to in ADS. For example, it is reasonable to assume that more novel words are introduced in interaction with an infant than in conversation with another adult, and so a higher degree of VH in IDS than in ADS may be a side effect of this contextual difference rather than vowels overall being more hyperarticulated in IDS. As for the potential impact of VH on different aspects of language development, experimental studies in which it can be separated from other adaptive behaviors should be conducted. This study is followed up by investigating the relationship between infant vocalization complexity and parent VH on the level of conversational turns, rather than on a subject level, in order to be able to provide some information about potential causality (Marklund et al., accepted).

In conclusion, the phonetic complexity of 12-month-old’s vocalizations is correlated with the degree of articulatory adaptation toward hyperarticulation in their parents’ IDS. The findings are in line with previous research in terms of a positive relationship between VH in IDS and infant language skills, as well as in that they do not provide information about causality of the relationship.

## Data Availability Statement

Tabular data generated for this study are available at the Open Science Framework (https://osf.io/rc8v4/).

## Ethics Statement

The studies involving human participants were reviewed and approved by the Regional Ethics Review Board in Stockholm (2015/63-31). Written informed consent to participate in this study was provided by the adult participants and the infant participants’ legal guardian/next of kin.

## Author Contributions

EM, UM, and LG: study design, drafting of the manuscript, and critical revisions of the manuscript. UM and LG: data collection (part) and transcriptions. EM: data processing and analyses. All authors contributed to the article and approved the submitted version.

## Conflict of Interest

The authors declare that the research was conducted in the absence of any commercial or financial relationships that could be construed as a potential conflict of interest.

## Footnotes

1 The longitudinal study was part of the MINT-project (MAW 2011.0070, PI Gerholm).

2 Note, however, that this is the same group of parent-infant dyads as in the present study.
